# Evidence of questionable research practices in clinical prediction models

**DOI:** 10.1186/s12916-023-03048-6

**Published:** 2023-09-04

**Authors:** Nicole White, Rex Parsons, Gary Collins, Adrian Barnett

**Affiliations:** 1 Australian Centre for Health Services Innovation and Centre for Healthcare Transformation, School of Public Health and Social Work, Faculty of Health, Queensland University of Technology, Kelvin Grove, Queensland Australia; 2 Centre for Statistics in Medicine, Nuffield Department of Orthopaedics, Rheumatology & Musculoskeletal Sciences, University of Oxford, Oxford, UK

**Keywords:** Prediction model, Area under curve, Diagnosis, Prognosis, Hacking, Statistics, Receiver operating characteristic

## Abstract

**Background:**

Clinical prediction models are widely used in health and medical research. The area under the receiver operating characteristic curve (AUC) is a frequently used estimate to describe the discriminatory ability of a clinical prediction model. The AUC is often interpreted relative to thresholds, with “good” or “excellent” models defined at 0.7, 0.8 or 0.9. These thresholds may create targets that result in “hacking”, where researchers are motivated to re-analyse their data until they achieve a “good” result.

**Methods:**

We extracted AUC values from *PubMed* abstracts to look for evidence of hacking. We used histograms of the AUC values in bins of size 0.01 and compared the observed distribution to a smooth distribution from a spline.

**Results:**

The distribution of 306,888 AUC values showed clear excesses above the thresholds of 0.7, 0.8 and 0.9 and shortfalls below the thresholds.

**Conclusions:**

The AUCs for some models are over-inflated, which risks exposing patients to sub-optimal clinical decision-making. Greater modelling transparency is needed, including published protocols, and data and code sharing.

**Supplementary Information:**

The online version contains supplementary material available at 10.1186/s12916-023-03048-6.

## Background

Clinical prediction models estimate an individual’s risk of being diagnosed with a disease or experiencing a future health outcome [1, 2]. A clinical prediction model uses multivariable analysis methods to estimate the risk of experiencing an outcome based on individual-level variables, for example, a model predicting a patient’s risk of death after admission to intensive care using data from their medical history and test results [3].

Researchers are motivated to build clinical prediction models because of their potential to support decision-making. Clinical decisions can be based on the model’s estimated probabilities or risk categories defined by probability cut-points to give qualitative interpretations, e.g. low and high risk [4]. Decisions guided by model probabilities or categories may rule out low-risk patients to reduce unnecessary treatments or identify high-risk patients for additional monitoring.

The number of published clinical prediction models has increased in recent years. A validated search strategy in MEDLINE [5] shows that an average of 4200 publications related to clinical prediction modelling are now being published weekly (searched 20 January 2023).

Despite being a popular study design, clinical prediction models are often poorly executed. Factors driving poor model quality include inadequate sample sizes, inappropriate exclusions, poor handling of missing data, limited model validation, and inflated estimates of performance [6–11]. A review of prediction models for COVID-19 found only 7 of the 606 published models were potentially useful for practice [12]; other reviews have identified shortcomings in model development that introduce bias into model predictions [13, 14]. Design failings are often compounded by poor reporting, despite the availability of expert-led guidance to improve transparency [1, 15, 16].

Rigorous testing of clinical prediction models is essential before considering their use in practice [17]. A good prediction model will have a strong discrimination, which is a model’s ability to separate patients based on their estimated risk. The area under the receiver operating characteristic curve (AUC) is an overall measure of model discrimination. It the probability that a model predicts a higher risk for a randomly selected patient *with* the outcome of interest than a randomly selected patient *without* the outcome of interest [18]. If the model has good discrimination and gives estimated risks for all patients with the outcome that are higher than all patients without, then the AUC will be 1. If the model discrimination is no better than a coin toss, then the AUC will be 0.5. The AUC is also known as the AUROC, c-statistic for binary outcomes, and c-index for time-to-event outcomes.

Qualitative descriptors of model performance for AUC thresholds between 0.5 and 1 have been published, for example:

- “0.7 to 0.8 is considered acceptable, 0.8 to 0.9 is considered excellent, and more than 0.9 is considered outstanding” [19].
- “The area under the ROC curve (AUC) results were considered excellent for AUC values between 0.9 and 1, good for AUC values between 0.8 and 0.9, fair for AUC values between 0.7 and 0.8, poor for AUC values between 0.6 and 0.7 and failed for AUC values between 0.5 and 0.6” [20].
- “Areas under the curve (AUCs) of 0.6 to 0.7, 0.7 to 0.8, 0.8 to 0.9 and > 0.9 were considered acceptable, fair, good and excellent for discrimination, respectively” [21].

Additional examples are in Additional file 1. These thresholds have no clear origin, but they are likely used because they transform the AUC from a number into a qualitative rating of performance. The thresholds have no scientific basis and are arbitrarily based on digit preference, often occurring at 0.7, 0.8 and 0.9 [22]. Previous research has examined the labels applied to AUC values in 58 papers and recommended that AUC values should be presented without labels [23].

Thresholds may create targets that some researchers will strive to achieve. We hypothesised that some researchers have engaged in questionable research practices or “hacking” to create models with estimated AUCs that better commonly used thresholds, including (1) re-analysing data and creating multiple models to get an AUC value over a threshold and (2) selectively reporting the best AUC value from many models [24]. Assuming the AUC has multiple thresholds (0.7, 0.8 and 0.9), we expected the distribution of AUC values would be undulating rather than smooth, with excess values just above the thresholds. We describe some ways a prediction model can be “hacked” in Table 1, but note this is not an exhaustive list.

**Table 1:** Examples of how a clinical prediction model can be hacked to get a better AUC value that is likely to be over-inflated as the model is over-fitted. Some of these approaches create multiple results from which the best result can be selected, often without disclosing the multiple results. Some hacking may be unintentional as researchers believe they are following standard practice. Some approaches can be acceptable when combined with appropriate validation, but the number of models fitted should always be disclosed and should be pre-defined in a protocol or pre-registration [1]

- Selectively choosing data sets (from those that are available to the researcher) to build and evaluate a model
- Collecting more data until a desirable AUC value is reached [25, 26]
- Fitting multiple, potentially hundreds, of models based on subsets of potential predictors [26]
- Trialling different cut-points when dichotomising continuous predictors until a “good” AUC is achieved [27]
- Including predictors that are proxies of the outcome or that work via reverse causality, for example, using blood tests taken after the outcome
- Changing the outcome variable, for example to a proxy of the original diagnostic outcome [26]
- Trialling alternative methods for imputing missing data [26]
- Removing observations that are difficult to fit [25, 26]
- Trialling different modelling approaches, e.g. logistic regression models and classification trees [28]
- Rounding up an AUC value to pass a threshold, for example reporting 0.79 as 0.8 [25]
- Choosing the “best” random seed for split sample validation or a model’s hyper-parameters [29]
- Not using internal validation, so the model performance is evaluated in the same data used to develop the model

The use of thresholds when reporting results from statistical analysis is not new. Well-known examples include 0.05 for the statistical significance of hypothesis tests and an 80% power to justify sample size calculations [30]. Related research has examined the enormous excess of *p*-values just below the widely used 0.05 threshold, which is caused by multiple data dredging techniques, including re-analyses of data and selective reporting [26, 31–33]. Recent research has also shown the same problem for Cronbach’s alpha at the “acceptable” threshold of 0.7 [34].

## Methods

### Data extraction

We aimed to find abstracts that included an area under the curve value or the related c-index for survival and c-statistic for binary outcomes [35]. These estimates have a variety of names, including “area under the receiver operating characteristic curve” or the acronyms “AUC” and “AUROC”. We included all AUCs regardless of the study’s aim and therefore included model development and validation studies. We did not consider other commonly reported metrics for evaluating clinical prediction models.

We examined abstracts published in *PubMed* because it is a large international database that includes most health and medical journals. To indicate its size, there were over 1.5 million abstracts published on *PubMed* in 2022. The National Library of Medicine make the *PubMed* data freely and easily available for research. We downloaded the entire database in XML format on 30 July 2022 from https://ftp.ncbi.nlm.nih.gov/pubmed/baseline/.

We started with all the available *PubMed* data. Our exclusion criteria were as follows:

- Entries with an empty abstract or an abstract of 10 words or fewer
- Pharmacokinetic studies, which often use area under the curve statistics to refer to dosages and volumes that are unrelated to prediction models
- Meta-analyses or pooled analyses, as we were interested in original research
- Tutorial papers, as these may not report original findings

Our inclusion criterion was abstracts with one or more AUC values.

We created a text-extraction algorithm to find AUC values using the team’s expertise and trial and error. We validated the algorithm by randomly sampling 300 abstracts with a Medical Subject Heading (MeSH) of “Area under curve” that had an abstract available and quantifying the number of AUC values that were correctly extracted. We also examined randomly selected results from the algorithm that equalled the thresholds of 0.7, 0.8 or 1, with 300 abstracts per threshold examined. We report the validation in more detail in the results, but note here that the algorithm could not reliably extract AUC values that were exactly 1. AUC values equal to 1 were therefore excluded.

Challenges in extracting the AUC values from abstracts included the frequent use of long lists of statistics, including the sensitivity and specificity; unrelated area under the curve statistics from pharmacokinetic studies; references to AUC values as a threshold (e.g. “The AUC ranges between 0.5 and 1”); and the many different descriptors used, including “area under the curve”, “receiver operating characteristic curve”, and related acronyms.

AUC values reported as a percent were converted to 0 to 1. We removed any AUC values that were less than 0 or greater than or equal to 1.

We categorised each AUC value as a mean or the lower or upper limit of the confidence interval, for example, “0.704 (95% CI 0.603 to 0.806)” would be a mean, lower and upper limit, respectively.

For the specific examples from published papers in the results, we give the *PubMed* ID number (PMID) rather than citing the paper.

R version 4.2.1 was used for data extraction and analysis [36]. The code and analysis data are available online: https://github.com/agbarnett/area_under_curve [37].

### Statistical analysis

Our hypothesis was that there would be an excess of AUC values just above the thresholds 0.7, 0.8 and 0.9. To examine this, we used a histogram with bins of (lower, upper], with lower thresholds of 0, 0.01 to 0.99, and an upper threshold that was + 0.01 greater. For example, the bin of (0.69, 0.70] included every AUC greater than 0.69 and less than or equal to 0.70. We excluded AUCs with a decimal place of 1 (e.g. “0.8”), as these results would create spikes in the histogram that were simply due to rounding.

We do not know what the distribution of AUC values from the health and medical literature would look like if there was no AUC-hacking. However, we are confident that it should be relatively smooth with no inflexion points. An undulating distribution, especially near the thresholds (0.7, 0.8 and 0.9), would be a strong sign of AUC-hacking, potentially caused by re-analysing the data to get a more publishable but inflated AUC.

We estimated the shape of a smooth distribution using a natural spline with 4 degrees of freedom fitted using a Poisson distribution [38]. We created residuals by subtracting the observed counts from the smooth fit. A similar approach was used to identify departures from a smooth distribution for a large sample of Cronbach’s alpha statistics [34].

During data collection, we noted that many abstracts gave multiple AUC values from competing models. To examine the best model per abstract, we plotted the distribution using the highest AUC value per abstract. This subgroup analysis examined whether the best presented models were often just above the thresholds.

We used a subgroup analyses that used only AUC values from the results section of structured abstracts. This potentially increased the specificity of the extracted AUC values, as those from the introduction, methods and discussion sections were more likely to be general references to the AUC rather than results.

To investigate the role of publication bias, we used a subgroup analysis of only papers published in the journal *PLOS ONE* which welcomes “negative” results and does not select based on impact or novelty [39].

## Results

**Fig. 1:**
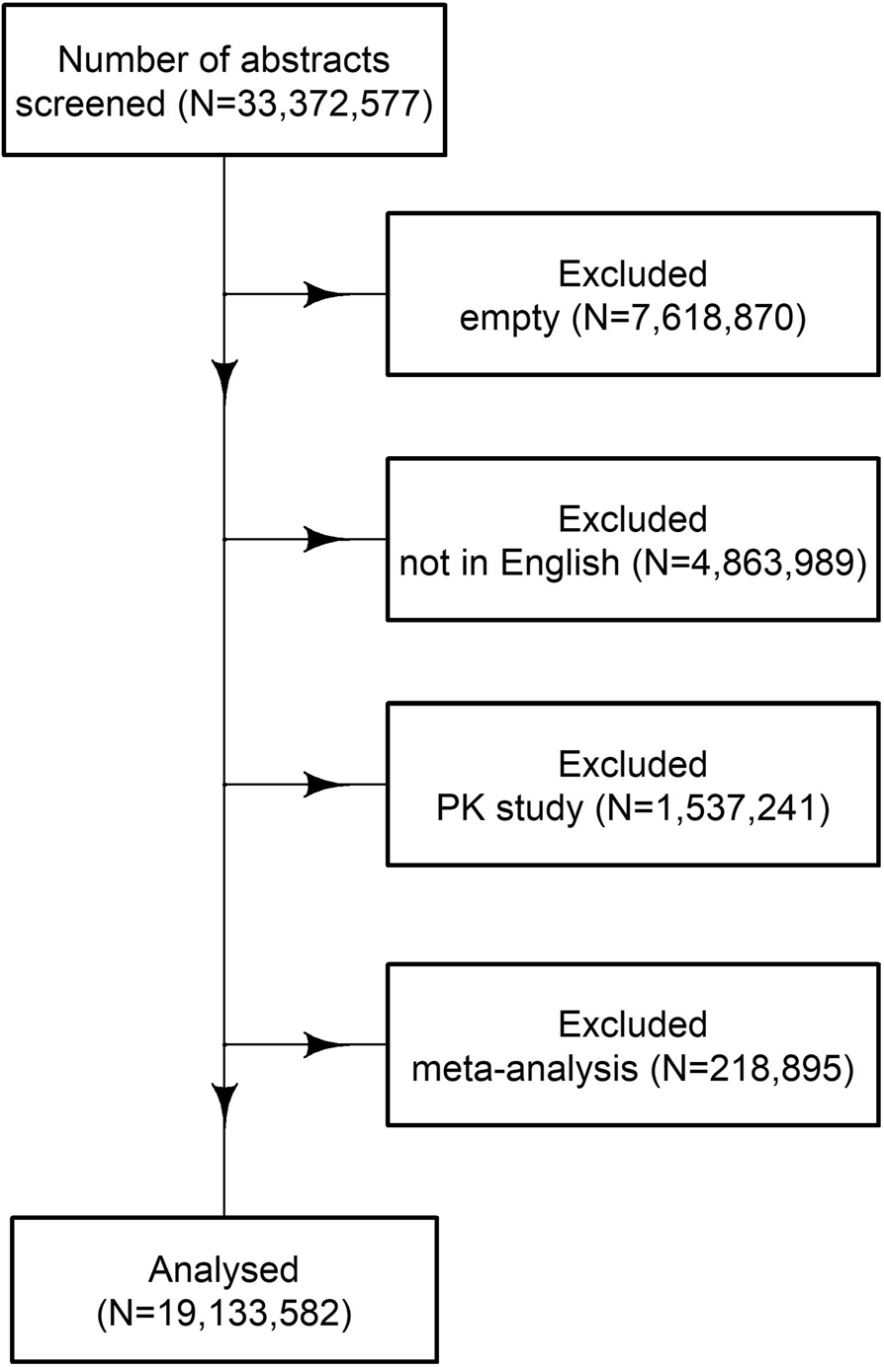
Flow chart of included abstracts. PK, pharmacokinetic

The flow chart of included abstracts is shown in Fig. 1.

The number of examined abstracts was over 19 million, and 96,986 (0.5%) included at least one AUC value. The use of AUC values has become more popular in recent years (see Additional file 2: Fig. S1). The median publication year for the AUC values was 2018, with first to third quartile of 2015 to 2018.

For abstracts with at least one AUC value, the median number of AUC values was 2, with a first to third quartile of 1 to 4 (see Additional file 3: Fig. S2). There was a long tail in the distribution of AUC values, with 1.1% of abstracts reporting 20 or more AUC values. These high numbers were often from abstracts that compared multiple models. The total number of included AUC values was 306,888. There were 92,529 (31%) values reported as lower or upper confidence limits and the remainder as means.

**Fig. 2:**
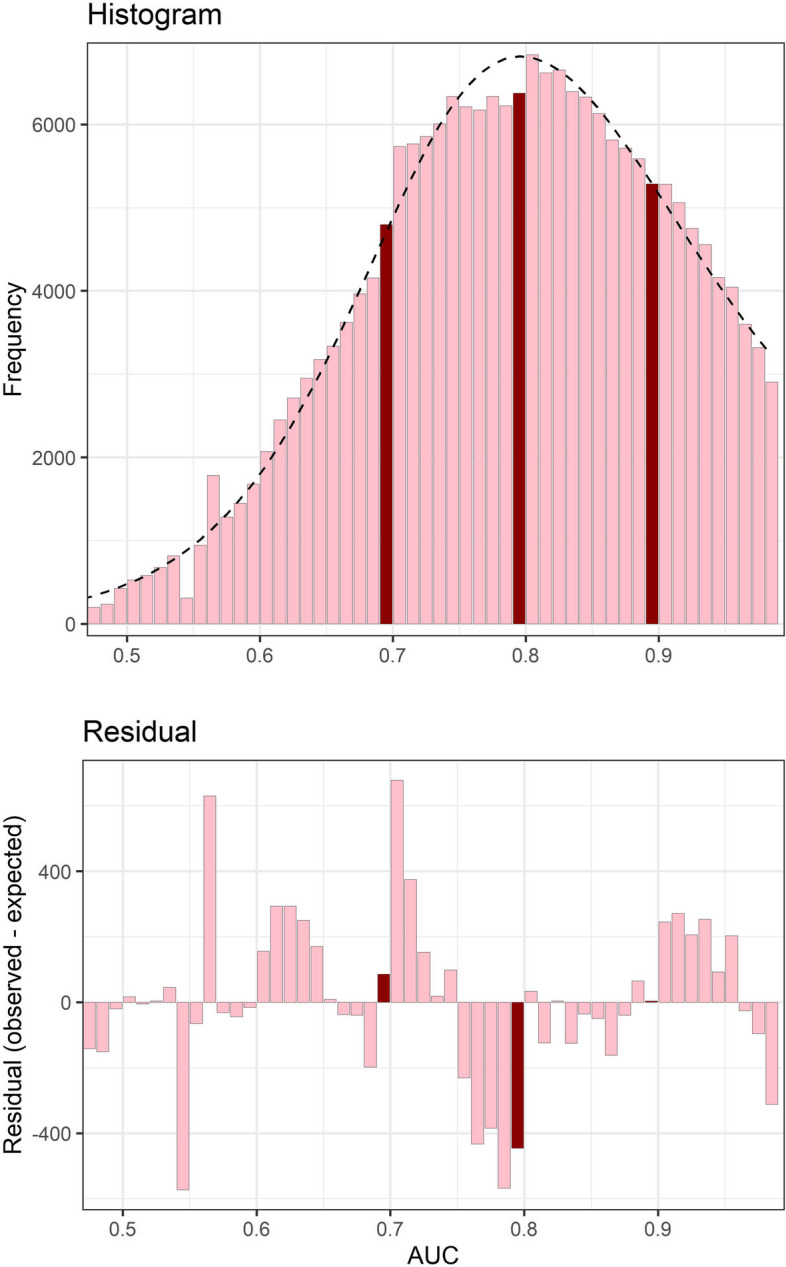
Histogram of AUC mean values (top panel) and residuals from a smooth fit to the histogram (bottom panel). The dotted line in the top panel shows the smooth fit

The distribution of AUC mean values (excluding confidence intervals) and residuals from the smoothed fit to the distribution are in Fig. 2. There are clear changes in the distribution around the thresholds of 0.7, 0.8 and 0.9. There is a large excess of AUC values just above 0.7, followed by a deficit before 0.8. There is a large jump in the number of AUCs just above 0.8 compared with (0.79, 0.80]. A similar excess is observed for AUC values just above 0.9. The frequencies in the histogram and residuals are worth noting, as they indicate thousands of unexpected results. There were 2106 (1.0%) AUC values presented to 1 decimal place that were excluded from the histogram.

**Fig. 3:**
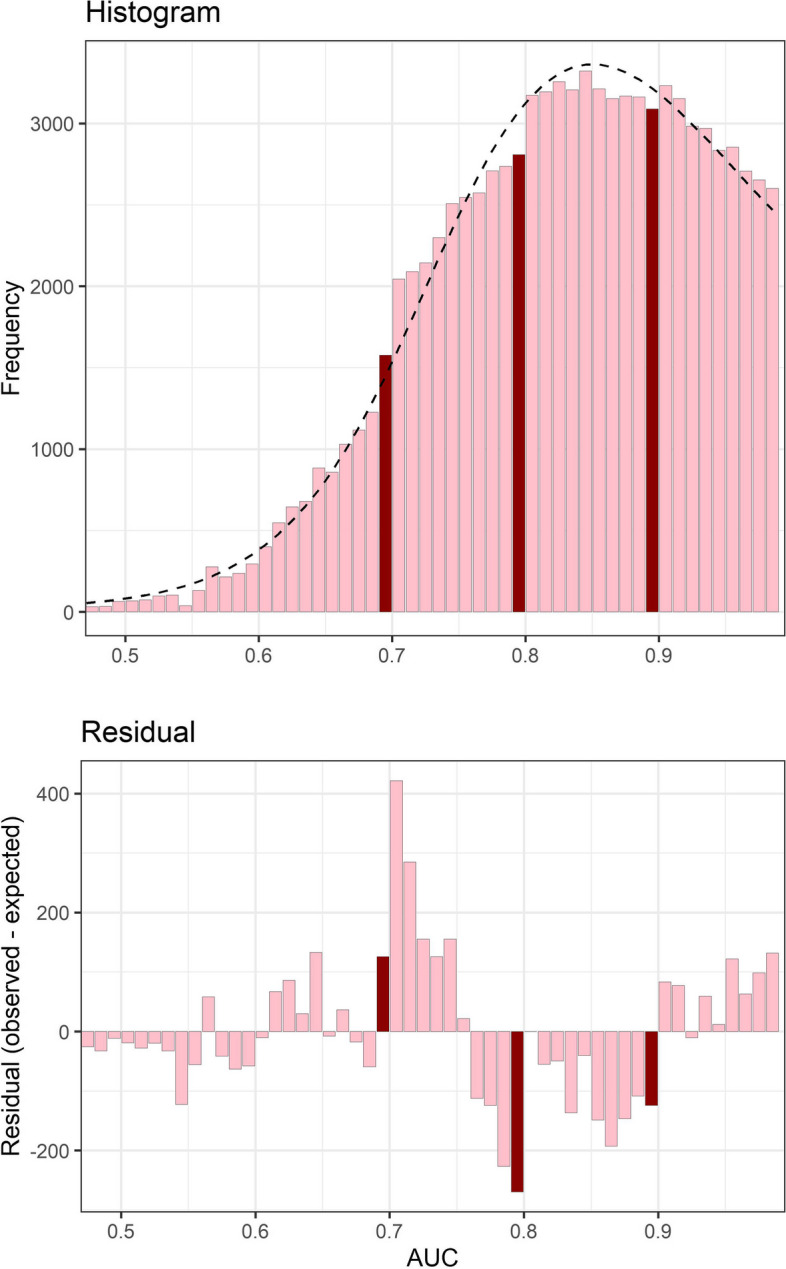
Histogram of the largest AUC mean value per abstract (top panel) and residuals from a smooth fit to the histogram (bottom panel). The dotted line in the top panel shows the smooth fit

The distribution from the largest AUC mean value per abstract excluding confidence intervals is shown in Fig. 3. The strong changes in the distribution at the thresholds observed in Fig. 2 remain.

The distribution for AUC values from the results section only is in Additional file 4: Fig. S3; the shape of the distribution is similar to that using all AUC values. The distributions for the lower and upper limits of the confidence interval were generally smoother than the mean; see Additional file 5: Fig. S4. However, there was a notable excess at (0.56, 0.57] for the lower interval.

The distribution for AUC values published in *PLOS ONE* show a similar pattern to the full sample, with many more AUC values just above the 0.8 threshold (see Additional file 6: Fig. S5).

### Validation

We validated our algorithm against 300 manually entered abstracts. For 192 abstracts, there were no AUC values in the abstract, and the algorithm correctly identified these absences for 93%. Some errors were because the abstracts were selected using the MESH term “Area under the curve” meaning that many pharmacokinetic studies were included. For the 108 abstracts with an AUC, the algorithm identified 98% correctly. See Additional file 7 for details.

For abstracts where either the algorithm or manual entry found one or more AUC values, we made a Bland–Altman plot of the number of AUC values extracted (see Additional file 7: Fig. S6). The 90% limits of agreement were – 2 to 0. On average, the algorithm missed more AUC values than the manual entry, a discrepancy that was generally due to non-standard presentations. We are comfortable with this difference, as we would rather lean towards missing valid AUC values than wrongly including invalid AUC values.

We used a regression model to examine differences in the AUC values extracted by the algorithm and manual entry. AUC values that were wrongly included by the algorithm were smaller on average than the AUC values that were correctly included. This is because the values extracted were often describing other aspects of the prediction model, for example, the Brier score, sensitivity and specificity.

The validation helped identify MESH terms that identified pharmacokinetic studies that were excluded from our main analysis. In a second validation, we manually checked 100 randomly sampled abstracts that the algorithm identified as not having an AUC statistic and another 100 randomly sampled abstracts that the algorithm identified as having an AUC statistic. All abstracts identified as not having an AUC statistic were correctly classified (95% confidence interval for negative predictive value: 0.964 to 1.000). All but one abstract identified as having an AUC statistic was correct (95% confidence interval for positive predictive value: 0.946 to 1.000).

In a third validation, we manually checked 300 AUC values extracted by our algorithm at the thresholds 0.7, 0.8 and 1. The results led us to exclude AUC values of 1, because these could not be accurately extracted. At the thresholds of 0.7 and 0.8, there were some errors due to qualitative descriptions of the thresholds instead of actual results. For comparison with the rounded thresholds (0.7 and 0.8), we manually checked 300 AUC values at 0.81. These checks had fewer errors as they were more often AUC values and were not being used as descriptive thresholds. We do not believe that the errors undermine our main point about AUC-hacking. The greater errors at the thresholds mean that the numbers at 0.7, 0.8 and 0.9 in Fig. 2 should likely be smaller, making for a larger gap between the thresholds and values exceeding the threshold, which would be stronger evidence of poor practice.

To investigate the excess at (0.56, 0.57], we manually extracted the AUC values from 300 abstracts where our algorithm found an AUC value of 0.57 and another 300 from 0.58 as a nearby comparison with no excess. The error proportions from the algorithm were relatively low (see Additional file 7: Table S3), indicating that the excess at 0.57 was not due to errors.

### Evidence of poor practice

Although documenting poor practice was not our goal, whilst reading abstracts, we encountered mistakes, poor reporting, and potential spin. Some papers had mistakes in their results, for example a 95% confidence interval for the AUC from 0 to 1 (PMID34795784) and upper limits over 1 (PMID34880677); others used excessive decimal places (PMID34456583). Some abstracts displayed a poor understanding of the AUC value, including authors declaring a “highest” AUC of 0.5 which is equivalent to a coin toss (PMID28708299); an upper confidence interval of an AUC value that was under 0.5, possibly because the disease label was mistakenly reversed (PMID28795781); the AUC being misinterpreted as a direct measure of sensitivity or specificity (PMID34674968); and the AUC being misinterpreted as a regression coefficient (PMID19410507). There were instances of potential spin [11, 40], with relatively low AUC values under 0.75 described as “excellent” (PMID35222547).

## Discussion

P-hacking has been observed in large parts of the literature [31, 33, 41, 42], so it is disappointing but not surprising to see hacking in AUC values. The discontinuities in the AUC distribution around the thresholds of 0.7, 0.8 and 0.9 shown by our analysis are not as egregious as the previously observed *p*-value discontinuity at 0.05. This is likely because AUC-hacking is spread over multiple thresholds, and because the *p*-value is often the most important statistic to some authors, as demonstrated by the verbal gymnastics applied to “non-significant” *p*-values [43].

There was a surprising shortfall of AUC values at (0.54, 0.55] and excess at (0.56, 0.57]. This pattern could also be due to hacking, as estimates up to 0.55 could be viewed by some researchers as too close to 0.5, indicating model predictions that are no better than random (values under 0.55 would be presented as 0.5 if rounded to one decimal place). This would explain the distinct lack of lower confidence limits at (0.54, 0.55] (Additional file 5: Fig. S4), as some researchers would be unhappy with a confidence interval that was not statistically significant when compared with the null hypothesis at 0.5. Multiple re-analyses options are available to tweak the lower confidence limit over the threshold, including adding predictors to the model or removing attested outliers (see Table 1) [44].

The implications of hacked analyses for the literature and evidence-based medicine is that some clinical prediction models will have less utility for health systems than promised. This is potentially serious if models have been translated into practice based on “excellent” AUC values that were hacked. Decisions about patient care will be compromised, with potentially missed diseases and unnecessary interventions. Hacking likely explains some of the reduction in model performance when published prediction models are externally validated [45], with the inflated AUCs values regressing to the mean.

Hacking may be lessened by using protocols, analysis plans, and registered reports [46, 47]. However, the uptake of registered reports has been modest, and protocols do not completely prevent important changes to the analysis [48–50]. Despite this poor uptake and practice, it is possible that protocols and registered reports will be an important part of future best practice, and they can help build trust in research, together with data and code sharing [51].

Our results indicate that some researchers have prioritised reporting a “good” result in their abstract that will help them publish their paper. By doing so, the wider issues of what is needed to produce a high-quality prediction model are downplayed. An AUC value alone cannot determine if a model is “acceptable” or “excellent”. As a measure of model discrimination, the AUC represents just one aspect of prediction model performance. Other important aspects include the model’s calibration, the costs and implications of false negatives and false positives, and whether a model is worthwhile for practice [52–56].

We found evidence that some researchers do not understand the AUC value, with errors in the presentation and interpretation of the AUC. Researchers have easy access multiple software tools to create AUC values, but may be unwilling to spend time learning the theory that underpins prediction models, leaving them with a poor understanding of a model’s limitations [57].

Evidence of hacking in practice is available from recent surveys which have reported relatively high instances of researchers engaging in questionable practices and fraud. A survey of Australian researchers reported many were aware of instances where colleagues had made up data (10%), altered data (8%), selectively excluded data (27%) or trialled iterative statistical analysis until finding a model that yielded a “significant” result (45%) [58]. A survey of Dutch researchers reported 8% admitted to falsifying or manipulating data [59]. A survey of US statisticians reported that 22% had been asked in the last 5 years to remove or alter data to better support the hypothesis, and 48% had been asked to stress only the “significant” findings [60]. The widespread use of these poor practices creates a biased evidence base and is misinforming health policy.

### Limitations

We did not examine other commonly reported performance metrics used to evaluate clinical prediction model performance. It is possible that values such as model sensitivity and specificity may also be influenced by “acceptable” thresholds.

We only used AUC values given in abstracts and did not examine the full text. Some papers may have only presented their best results in the abstract and given a more complete picture in the full text. However, an analysis of *p*-values found that the distribution was similarly blighted by p-hacking when using *p*-values from the abstract or full text [32], and study of spin in prediction models found its occurrence was similar in the abstract and full text [11]. It is likely that the highest AUC value presented in the abstract is also the highest in the full text, so the “best” model would be captured in the abstract, and the “best” AUC value is the one most likely to be created by hacking.

In addition to hacking, publication bias likely also plays a role in the selection of AUC values, with higher values more likely to be accepted by peer reviewers and journal editors. Our subgroup analysis of *PLOS ONE* abstracts (Additional file 6: Fig. S6) provides some evidence that the “hacking” pattern in AUC values is due to author behaviour not journal behaviour.

We used an automated algorithm that provided a large and generalisable sample but did not perfectly extract all AUC values. In particular, we were not able to reliably extract AUC values of 1, and this is an important value as it is the best possible result and could be a target for hacking. We believe that the errors and exclusions in the data are not large enough to change our key conclusion, which is that AUC-hacking has occurred.

## Conclusions

Clinical prediction models are growing in popularity, likely because of increased patient data availability and accessible software tools to build models. However, many published models have serious flaws in their design and presentation. Our results show another serious issue, as the AUCs for some models have been over-inflated, and we believe this is due to hacking. Publishing overly optimistic models risks exposing patients to sub-optimal clinical decision-making. An urgent reset is needed in how clinical prediction models are built, validated and peer-reviewed. Actionable steps towards greater transparency are as follows: the wider use of protocols and registered reports, following expert reporting guidance, and increased data and code sharing.

### Supplementary information

**Additional file 1.** Examples of qualitative descriptors for AUC thresholds.

**Additional file 2:** **Figure S1.** Number and proportion of abstracts with at least one AUC value over time.

**Additional file 3:** **Figure S2.** Bar chart of the number of AUC values per abstract.

**Additional file 4:** **Figure S3.** Distribution of AUC values and residuals from a smooth fit to the distribution using only AUC values that were in the results section of the abstract.

**Additional file 5:** **Figure S4.** Histograms of AUC values that were lower or upper confidence limits and residuals from a smooth fit to the histograms.

**Additional file 6:** **Figure S5.** Subgroup analysis of AUC values from the journal *PLOS ONE*.

**Additional file 7:** **Figure S6.** Bland–Altman plot of the difference in the number of AUC values per abstract extracted manually and by the algorithm. **Figure S7.** Box-plots of AUC values grouped by whether they were extracted by the algorithm or manual-check only, or by both. **Table S1.** Estimates from a linear regression model examining the differences in AUC values extracted by the algorithm and manual checking. **Figure S8.** Proportion of correct AUC values from the algorithm for four selected AUC values. **Table S2.** Proportion of correct AUC values from the algorithm for two selected AUC values.

## Data Availability

The code and analysis data are available online: https://github.com/agbarnett/area_under_curve, including code used to read in and wrangle the publicly available data hosted by *PubMed*. The *PubMed* data are available here https://dtd.nlm.nih.gov/ncbi/pubmed/doc/out/190101/index.html along with the attributes to the accessible data. The specific dataset used in this study can be downloaded by file transfer protocol ftp://ftp.ncbi.nlm.nih.gov/pubmed/baseline and can be accessed by a file explorer.

## Abbreviations

AUC, AUROC: Area under the receiver operating characteristic curve
CI: Confidence interval
PK: Pharmacokinetic
ROC: Receiver operating characteristic
