# Supplementary material for: Evidence of questionable research practices in clinical prediction models
Source: BMC Med. 2023 Sep 4;21:339. doi: 10.1186/s12916-023-03048-6 (PMC10478406; doi:10.1186/s12916-023-03048-6)
Supplement: Supplementary file 1 — Additional file 1. Examples of qualitative descriptors for AUC thresholds. [file 12916_2023_3048_MOESM1_ESM.pdf]

Additional file 1: Examples of qualitative descriptors for AUC thresholds

- “A model having c greater than roughly .8 has some utility in predicting the responses of individual subjects.” [1]
- “An AUC value of more than 0.9 was considered outstanding and 0.8–0.9 as excellent.” PMID35537614
- “An AUC of 0.5 suggests no discrimination, 0.7–0.8 is considered acceptable, more than 0.8–0.9 is considered excellent, and more than 0.9 is considered outstanding.” PMID34793687
- “A value of .5 denotes no prediction accuracy, 1 denotes perfect accuracy and heuristically, .6 to .7 can be regarded as weak, .7 to .85 as moderate, and more than .85 as good, although the convention varies considerably by discipline and analysis goal.” PMID23778288
- “Usually, good values of AUC start from .75, but again, this depends on the problem and looking at absolute values is generally not helpful. You’d rather use it to compare models. If your model has an AUC of 0.57 that means there’s likely no signal in your data.” [2]
- “Generally speaking, when the AUC value of a model is at the range of 0.7–0.8, the prediction ability of the model is superior. When the AUC value is at the range of 0.8–0.9, the prediction ability of the model is very good. Through the SMOTE-Bagged Tree algorithm, the AUC value obtained by our model is 0.80, which proves that our model has a proper prediction ability, and can be used for early breast cancer patients.” [3]
- “So, what area under the ROC curve describes good discrimination? Unfortunately there is no “magic” number, only general guidelines. In general, we use the following rule of thumb: ROC = 0.5 This suggests no discrimination, so we might as well flip a coin. $0.5 < \text{ROC} < 0.7$ We consider this poor discrimination, not much better

than a coin toss. $0.7 \leq$ ROC < 0.8 We consider this acceptable discrimination. 0.8 $\leq$ ROC < 0.9 We consider this excellent discrimination. ROC ≥ 0.9 We consider this outstanding discrimination.” [4]

- “There are several scales for AUC value interpretation but, in general, ROC curves with an AUC $\leq$0.75 are not clinically useful and an AUC of 0.97 has a very high clinical value, correlating with likelihood ratios of approximately 10 and 0.1.” [5]

The PMID is the *PubMed* number. These examples were found by convenience, not from a systematic search.

References

[1] Harrell FE. Regression Modeling Strategies: With Applications to Linear Models, Logistic Regression, and Survival Analysis. Springer Series in Statistics. Springer New York; 2013.

[2] Revert F.: The proper way to use Machine Learning metrics. Available from: https://towardsdatascience.com/the-proper-way-to-use-machine-learning-metrics-4803247a2578.

[3] Liu C, Zhao Z, Gu X, Sun L, Chen G, Zhang H, et al. Establishment and Verification of a Bagged-Trees-Based Model for Prediction of Sentinel Lymph Node Metastasis for Early Breast Cancer Patients. Front Oncol. 2019;9. https://doi.org/10.3389/fonc.2019.00282.

[4] Hosmer DW, Lemeshow S, Sturdivant RX. Applied Logistic Regression. Wiley Series in Probability and Statistics. Wiley; 2013.

[5] Fan J, Upadhye S, Worster A. Understanding receiver operating characteristic (ROC) curves. CJEM. 2006;8(1):19–20. https://doi.org/10.1017/S1481803500013336.
