# Supplementary material for: Evidence of questionable research practices in clinical prediction models
Source: BMC Med. 2023 Sep 4;21:339. doi: 10.1186/s12916-023-03048-6 (PMC10478406; doi:10.1186/s12916-023-03048-6)
Supplement: Supplementary file 2 — Additional file 2: Figure S1. Number and proportion of abstracts with at least one AUC value over time. [file 12916_2023_3048_MOESM2_ESM.pdf]

Additional file 2: Trends over time in the use of AUC

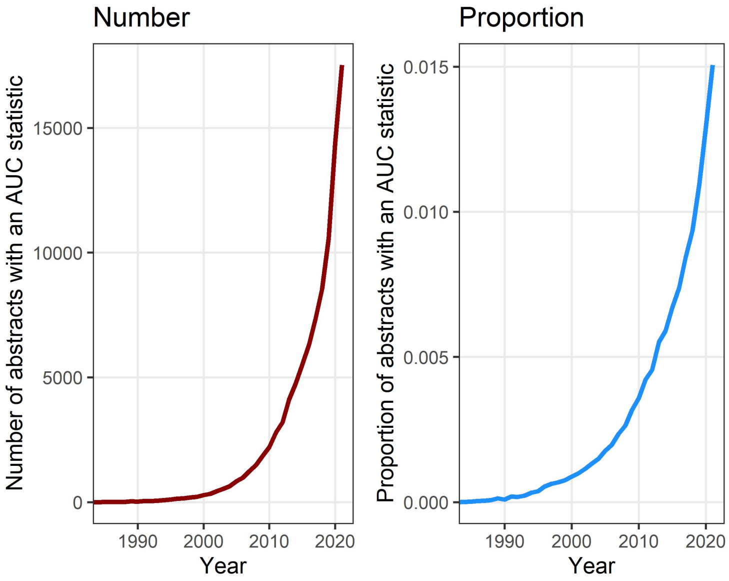

Fig. S1 Number and proportion of abstracts with at least one AUC value over time. The year axis starts at 1985.
