# Supplementary material for: Evidence of questionable research practices in clinical prediction models
Source: BMC Med. 2023 Sep 4;21:339. doi: 10.1186/s12916-023-03048-6 (PMC10478406; doi:10.1186/s12916-023-03048-6)
Supplement: Supplementary file 3 — Additional file 3: Figure S2. Bar chart of the number of AUC values per abstract. [file 12916_2023_3048_MOESM3_ESM.pdf]

Additional file 3: AUC values per abstract

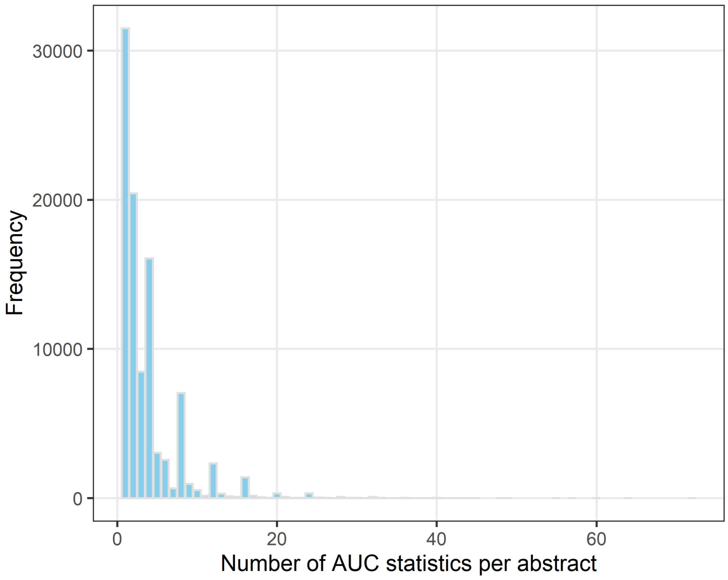

Fig. S2 Bar chart of the number of AUC values per abstract. For abstracts with at least one AUC.
