# Supplementary material for: Evidence of questionable research practices in clinical prediction models
Source: BMC Med. 2023 Sep 4;21:339. doi: 10.1186/s12916-023-03048-6 (PMC10478406; doi:10.1186/s12916-023-03048-6)
Supplement: Supplementary file 4 — Additional file 4: Figure S3. Distribution of AUC values and residuals from a smooth fit to the distribution using only AUC values that were in the results section of the abstract. [file 12916_2023_3048_MOESM4_ESM.pdf]

Additional file 4: Sensitivity analysis: AUC values that were in the results sections of structured abstract

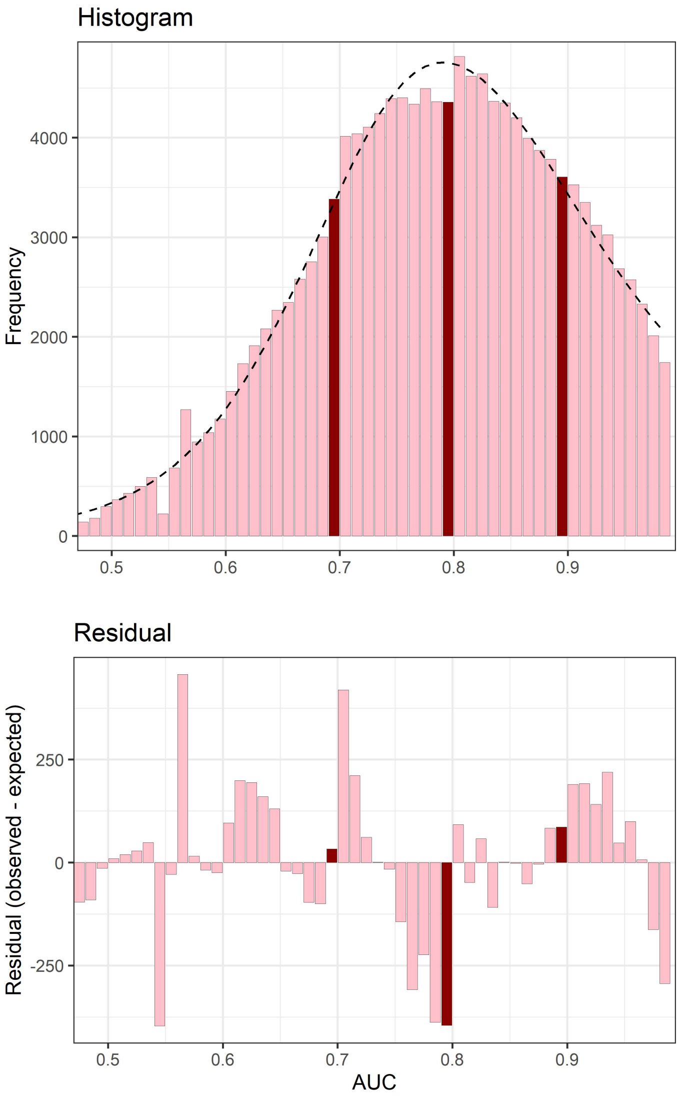

Fig. S3 Distribution of AUC values (top panel) and residuals from a smooth fit to the distribution (bottom panel). Using only AUC values that were in the results section of the abstract.
