# Supplementary material for: Evidence of questionable research practices in clinical prediction models
Source: BMC Med. 2023 Sep 4;21:339. doi: 10.1186/s12916-023-03048-6 (PMC10478406; doi:10.1186/s12916-023-03048-6)
Supplement: Supplementary file 5 — Additional file 5: Figure S4. Histograms of AUC values that were lower or upper confidence limits and residuals from a smooth fit to the histograms. [file 12916_2023_3048_MOESM5_ESM.pdf]

## Additional file 5: AUC values from the lower and upper confidence limits

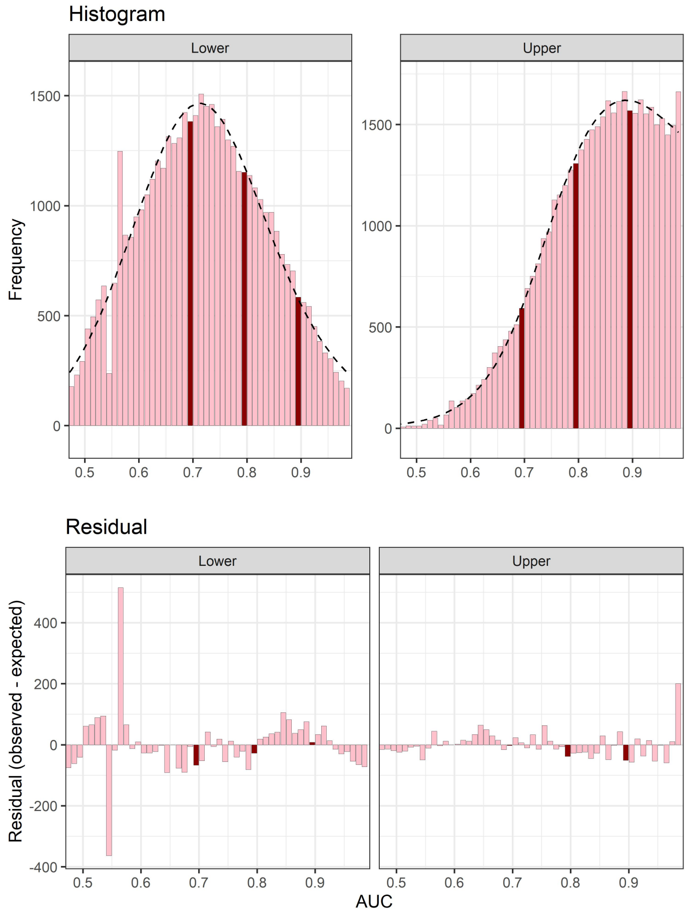


**Fig. S4** Histograms of AUC values that were lower or upper confidence limits (top panel) and residuals from a smooth fit to the histograms (bottom panel). The dotted line in the top panel shows the smooth fit.

There was an excess in models with an upper confidence limit of 0.98 and above. This may be due to studies with small sample sizes and a large uncertainty in the AUC value. There is a surprising excess of models with a lower confidence limit of (0.56, 0.57] and corresponding shortfall at (0.54, 0.55].
