# Supplementary material for: Evidence of questionable research practices in clinical prediction models
Source: BMC Med. 2023 Sep 4;21:339. doi: 10.1186/s12916-023-03048-6 (PMC10478406; doi:10.1186/s12916-023-03048-6)
Supplement: Supplementary file 6 — Additional file 6: Figure S5. Subgroup analysis of AUC values from the journal PLOS ONE. [file 12916_2023_3048_MOESM6_ESM.pdf]

Additional file 6: Mean AUC values from abstracts published in PLOS ONE

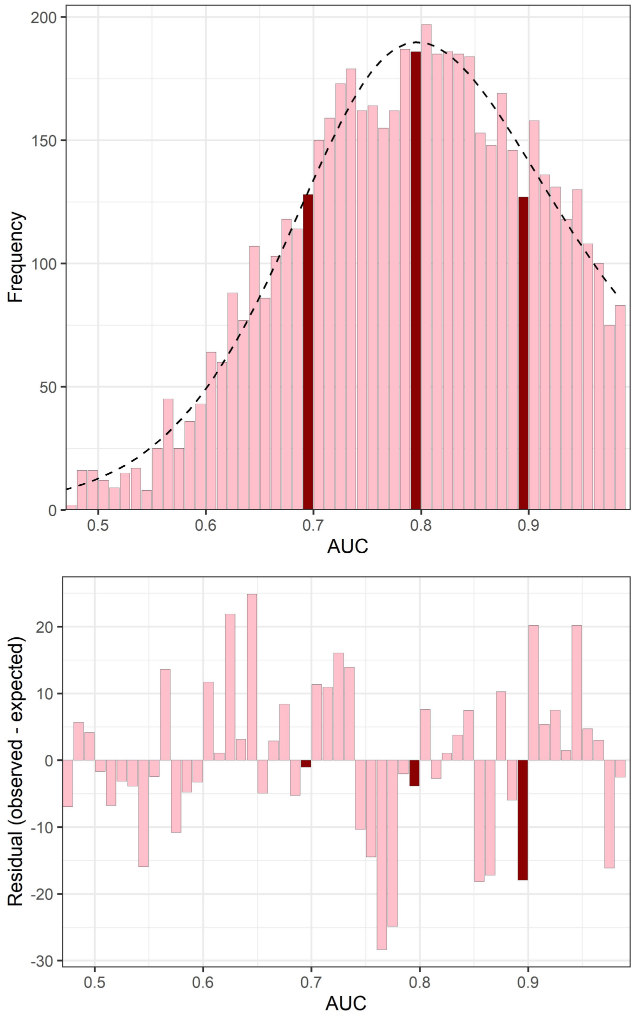

Fig. S5 Subgroup analysis of AUC values from the journal PLOS ONE. Histogram of AUC mean values (top panel) and residuals from a smooth fit to the histogram (bottom panel). The dotted line in the top panel shows the smooth fit.

The total number of AUC values in this subgroup analysis is 8,575 from 2,551 abstracts.
