# Supplementary material for: Evidence of questionable research practices in clinical prediction models
Source: BMC Med. 2023 Sep 4;21:339. doi: 10.1186/s12916-023-03048-6 (PMC10478406; doi:10.1186/s12916-023-03048-6)
Supplement: Supplementary file 7 — Additional file 7: Figure S6. Bland–Altman plot of the difference in the number of AUC values per abstract extracted manually and by the algorithm. Figure S7. Box-plots of AUC values grouped by whether they were extracted by the algorithm or manual-check only, or by both. Table S1. Estimates from a linear regression model examining the differences in AUC values extracted by the algorithm and manual checking. Figure S8. Proportion of correct AUC values from the algorithm for four selected AUC values. Table S2. Proportion of correct AUC values from the algorithm for two selected AUC values. [file 12916_2023_3048_MOESM7_ESM.pdf]

Additional file 7: Validation of the algorithm to extract AUC values

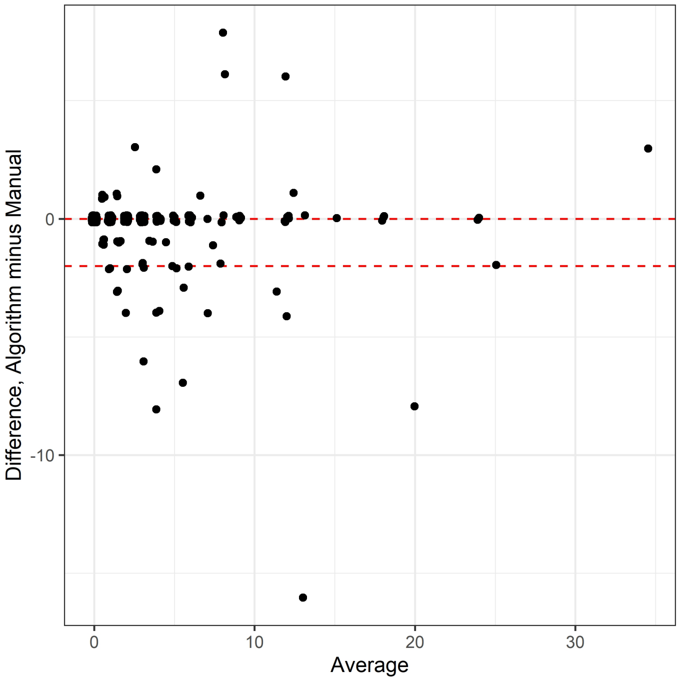

Fig. S6 Bland–Altman plot of the difference in the number of AUC values per abstract extracted manually and by the algorithm

The 90% limits of agreement for the number of AUC values per abstract were −2 to 0, so the algorithm leans towards missing AUC values. One of the large disagreements, where the algorithm missed many values, was a tutorial study (PMID33977829), and we subsequently excluded these studies. The algorithm sometimes included the wrong values, such as the area under the precision-recall curve (PMID33706377). Other large differences occurred when an abstract included lots of AUC values that were intertwined in the text and difficult to separate (PMID35945782).

The box-plots of AUC values shows that the values wrongly included by the algorithm were smaller on average than those that were included by the algorithm and manual check. The estimates from a linear regression model are in Table S1, the

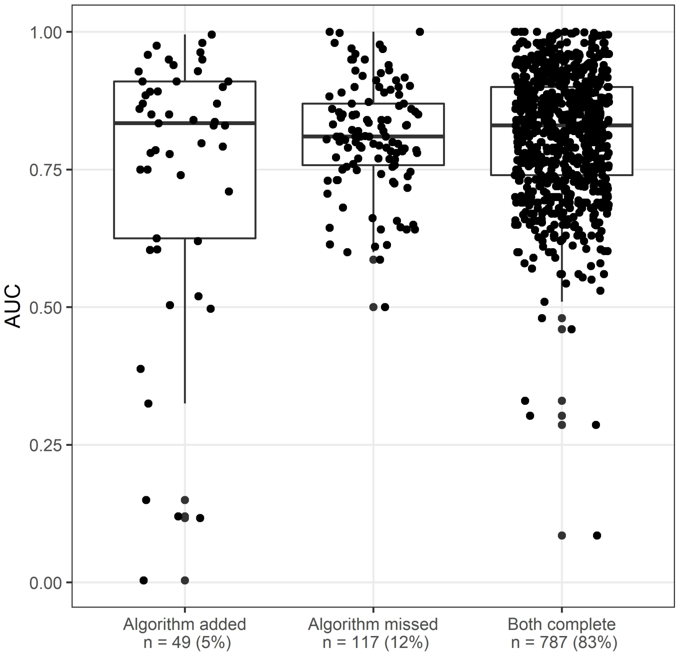

Fig. S7 Box-plots of AUC values grouped by whether they were extracted by the algorithm or manual-check only, or by both

reference category is those included by the algorithm and manual check. The model estimates show the bias the values wrongly added by the algorithm.

Table S1 Estimates from a linear regression model examining the differences in AUC values extracted by the algorithm and manual checking

		95% confidence interval	
	Estimate	Lower	Upper
Intercept	0.815	0.806	0.823
Algorithm wrongly added	–0.074	–0.109	–0.039
Algorithm wrongly missed	–0.016	–0.039	0.007

Checking AUCs that are used as thresholds

AUCs at 0.7, 0.8, and 0.9 may be presented as thresholds rather than results, for example, "with most area under the curve exceeding 0.8" (PMID26649802) and "The AUCs were always higher than 0.7" (PMID34847490). These thresholds would

artificially inflate the numbers at the thresholds. To investigate this we randomly sampled 300 abstracts where the algorithm gave AUCs of 0.7, 0.8 and 1. For comparison we also sampled 300 abstracts at 0.81 as this was unlikely to be used as a threshold. There were three raters, with 100 values per rater per AUC.

The proportion correct for the four AUC values are shown in the table below. The numbers assessed are just under 300 for some AUC values because there were some duplicates.

Table S2 Proportion of correct AUC values from the algorithm for four selected AUC values

				Confidence interval	
AUC	Assessed	Correct	Proportion	Lower	Upper
0.7	299	279	0.933	0.899	0.956
0.8	299	265	0.886	0.845	0.918
0.81	300	289	0.963	0.935	0.980
1	291	196	0.674	0.618	0.725

The algorithm fails to accurately capture AUC values that are 1, with a proportion correct of just 0.674. This is because 1 is commonly used in: labels (e.g., "group 1"), other statistics, and as a threshold. We therefore decided to exclude 1 from the results.

The algorithm works better for the AUC values of 0.7 and 0.8 than 1. However, there were still errors, which included when the AUC value was given as a threshold rather than a result.

As a comparison to 0.8 we examined the accuracy of 0.81, and the proportion correct by the algorithm is much higher as this value was never used as a threshold.

Investigating the excess of AUC values at (0.56, 0.57]

We examined 300 randomly sampled AUC values where the algorithm gave 0.57 because of the unusual excess of AUC values shown in Figure 2 and Additional file 5 Fig. S4. We examined 300 randomly sampled values of 0.58 as a nearby comparison to 0.57. The proportion correct for 0.57 and 0.58 were similar and both relatively

high (see table below). This indicates that the excess at (0.56, 0.57] is not due to an error in the algorithm.

Table S3 Proportion of correct AUC values from the algorithm for two selected AUC values

AUC	Assessed	Correct	Proportion	Confidence interval Lower	Upper
0.57	284	277	0.975	0.949	0.988
0.58	293	277	0.945	0.913	0.966

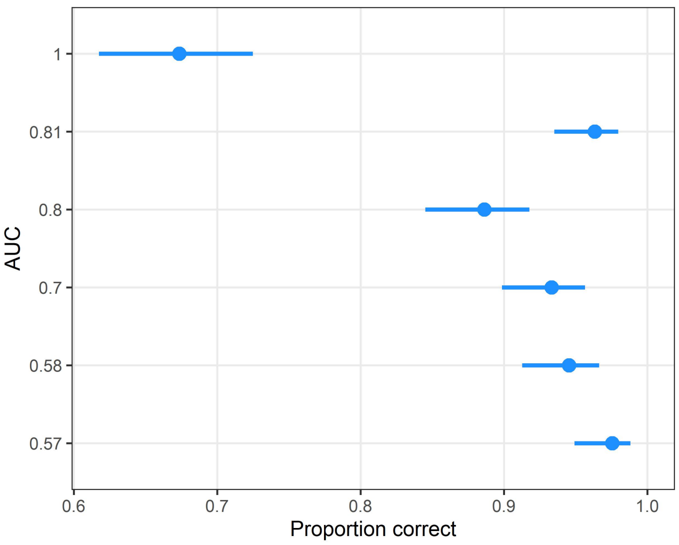

Fig. S8 Proportion of correct AUC values from the algorithm for six selected AUC values. Mean proportion (dot) and 95% confidence interval (horizontal line).
